# Self-rolling film for *in vivo* drug delivery to limit colonic inflammation induced after radiation exposure

**DOI:** 10.1016/j.isci.2026.117453

**Published:** 2026-09-03

**Authors:** Diego Amarante-Silva, Christelle Demarquay, Benjamin Nottelet, Mathilde Grosjean, Sidzigui Ouedraogo, Fabien Milliat, Karine Anselme, Arnaud Ponche, Noëlle Mathieu

**Affiliations:** 1 Nuclear Safety and Radiation Protection Authority (ASNR), Department of Radiobiology and Regenerative Medicine, Radiobiology of Medical Exposure Laboratory, LRMed, Fontenay-aux-Roses, France; 2 Polymer for Health and Biomaterials, IBMM, University Montpellier, CNRS, ENSCM, Montpellier, France; 3 Department of Pharmacy, Nîmes University Hospital, 30900 Nimes, France; 4 Institut de Science des Matériaux de Mulhouse (IS2M), CNRS/Université de Haute Alsace (UHA), UMR, 7361 Mulhouse, France

**Keywords:** radiotherapy side effects, self-rolling film, local treatment, anti-inflammatory drug delivery, colonoscopy, *in vivo* effect

## Abstract

Radiotherapy is a cornerstone of cancer treatment; however, despite major technological advances, radiation-induced injury to surrounding healthy tissues remains a significant clinical challenge. Here, we developed a preclinical rat model of radiation proctitis using localized, fractionated colorectal irradiation followed by comprehensive histopathological characterization. To enable local drug delivery while minimizing systemic exposure, we engineered a self-rolling bilayer PEG-PLA film loaded with the anti-inflammatory drug prednisolone. The biomaterial was optimized for endoscopic implantation into the irradiated colorectum, providing a minimally invasive therapeutic approach. Local administration of prednisolone significantly reduced the secretion of pro-inflammatory cytokines, including IL-1β, within the colonic mucosa, demonstrating the efficacy of the device in attenuating radiation-induced inflammation. Beyond prednisolone delivery, this adaptable platform could support localized administration of a broad range of therapeutic agents, including biologics or other compounds whose systemic use is limited by toxicity, poor tolerability, or high cost.

## Highlights

• Histopathological and innate immune profiling after colorectal radiation injury
• Developed a PEG-PLA film for local anti-inflammatory drug, prednisolone, delivery
• Engineered a self-rolling bilayer optimized for endoscopic implantation
• Local prednisolone reduces inflammatory mediators in irradiated colonic tissues

## Graphical abstract

**Figure:**
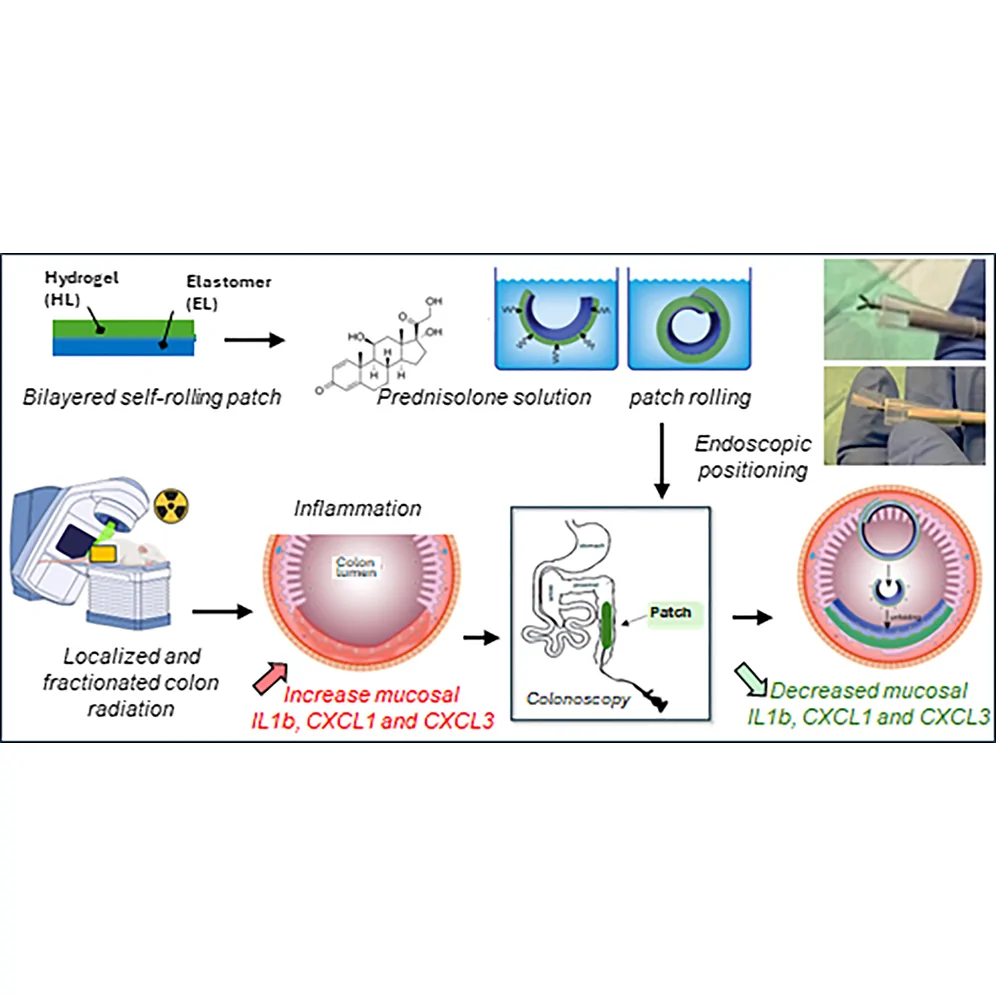

## Teaser

Drug delivery system; Radiation biology; Biomaterials

## Introduction

For over a century, radiotherapy (RT) has stood as a cornerstone of cancer treatment and remains a therapeutic mainstay for more than 50% of all cancer patients. Over the years, RT has undergone continuous technological refinement, including advancements in imaging for precise tumor delineation, enhanced ballistic accuracy, optimized dose delivery protocols, and, most recently, the introduction of particle therapy—all of which have collectively contributed to improved tumor regression. Nevertheless, even with these cutting-edge innovations, it remains impossible to entirely prevent the exposure of healthy tissues to clinically significant incidental doses of radiation. Such exposure can result in substantial long-term morbidity, thereby compromising patients’ quality of life. Over the past several decades, cancer mortality rates have steadily declined, while the number of cancer survivors has nearly tripled.^1^^,^^2^ As the cohort of cancer survivors continues to expand, there is an urgent need to intensify efforts aimed at mitigating the adverse effects of RT.^3^

Approximately half of all cancer survivors have received treatment for abdominal or pelvic tumors. The symptoms of toxicity associated with abdomino-pelvic RT vary depending on the tumor’s location and typically include abdominal pain, vomiting, alternating episodes of constipation and diarrhea, and, in some cases, incontinence.^4^ Radiation proctitis, a frequent complication, is characterized by rectal inflammation that develops following radiation therapy. In some patients, symptoms may escalate to severe rectal bleeding, necessitating interventions, such as blood transfusions and endoscopic vessel cauterization using plasma argon or formalin. In the most severe cases, affecting 1%–2% of patients, the condition may progress to complete obstruction or fistulae.^5^

The pathophysiology of pelvic radiation disease remains incompletely understood. It is postulated to involve an initial phase of acute intestinal inflammation, which may either resolve spontaneously or progress to chronic inflammation, ultimately culminating in fibrosis.^6^ Radiation-induced tissue toxicities are likely multifactorial, dependent on the specific tissue affected, and evolve over time. To date, no definitive curative treatment exists for this condition.

Several irradiation protocols have been developed to model radiation proctitis and evaluate the efficacy of treatments, such as mesenchymal stromal cells, organoids from colon and PDGF-C.^7^^,^^8^^,^^9^^,^^10^ These models use a single high dose of irradiation, localized on the colon, to obtain severe radio-induced damage. To more closely mimic clinical conditions and study the physiopathology of radiation proctitis, fractionated irradiation protocol has been developed in mice.^11^^,^^12^ In this study, we developed a rat model of radiation proctitis to enable endoscopic access for local treatment delivery. Unlike mice, rats provide sufficient luminal diameter to perform repeated endoscopic procedures, making them more suitable for evaluating localized therapeutic strategies. This choice is particularly relevant because, in patients with bowel dysfunction, the adverse effects of long-term oral administration of active compounds can outweigh their therapeutic benefits. In cancer patients, systemic drug delivery may also interfere with anti-tumor treatments, especially when immunomodulatory pathways are targeted. These limitations have motivated interest in local drug administration, and several studies have already explored direct colonic delivery. For instance, a randomized clinical trial demonstrated the beneficial effects of glucocorticoids administered via enema before lesion appearance during the RT protocol. Although these findings are encouraging, they should be replicated in a multicenter study with longer-term patient follow-up.^13^

Given the large number of patients affected by colonic pathologies in Western countries and the potential significant benefits for patients, considerable research has focused on improving drug targeting for the colon.^14^^,^^15^^,^^16^^,^^17^ To enhance active substance retention in the colon, some studies have developed carriers with tropism toward inflamed colonic sites^18^ or a temperature-triggered *in situ* forming lipid gels for the topical treatment of colitis.^19^ However, in these systems, drug release is not directed toward the mucosal surface, leading to dilution of the active ingredient by luminal fluids and proteolytic digestion. Recent innovations in biomaterial technologies, particularly patches and microdevices, offer promising approaches for colonic therapy.^14^^,^^15^ In this study, we used an optimized self-rolling film loaded with prednisolone which can be easily deployed via colonoscopy in the rat colon. Prednisolone is a synthetic glucocorticoid widely used for its anti-inflammatory and immunosuppressive effects in inflammatory colonic diseases, such as ulcerative colitis and Crohn’s disease.^20^^,^^21^ We characterized the inflammatory response induced by a localized and fractionated irradiation protocol in a rat model. Furthermore, we provide *in vivo* proof of concept for the feasibility and the benefit of local colonic treatment using a self-rolling bilayer film composed of PEG-PLA (polyethylene glycol [PEG] and hydrophobic polylactic acid [PLA]) and impregnated with the anti-inflammatory drug prednisolone, which is endoscopically implanted in the irradiated colon.

## Results

### Colonic histological damage induced by localized and fractionated irradiation

**Figure 1:**
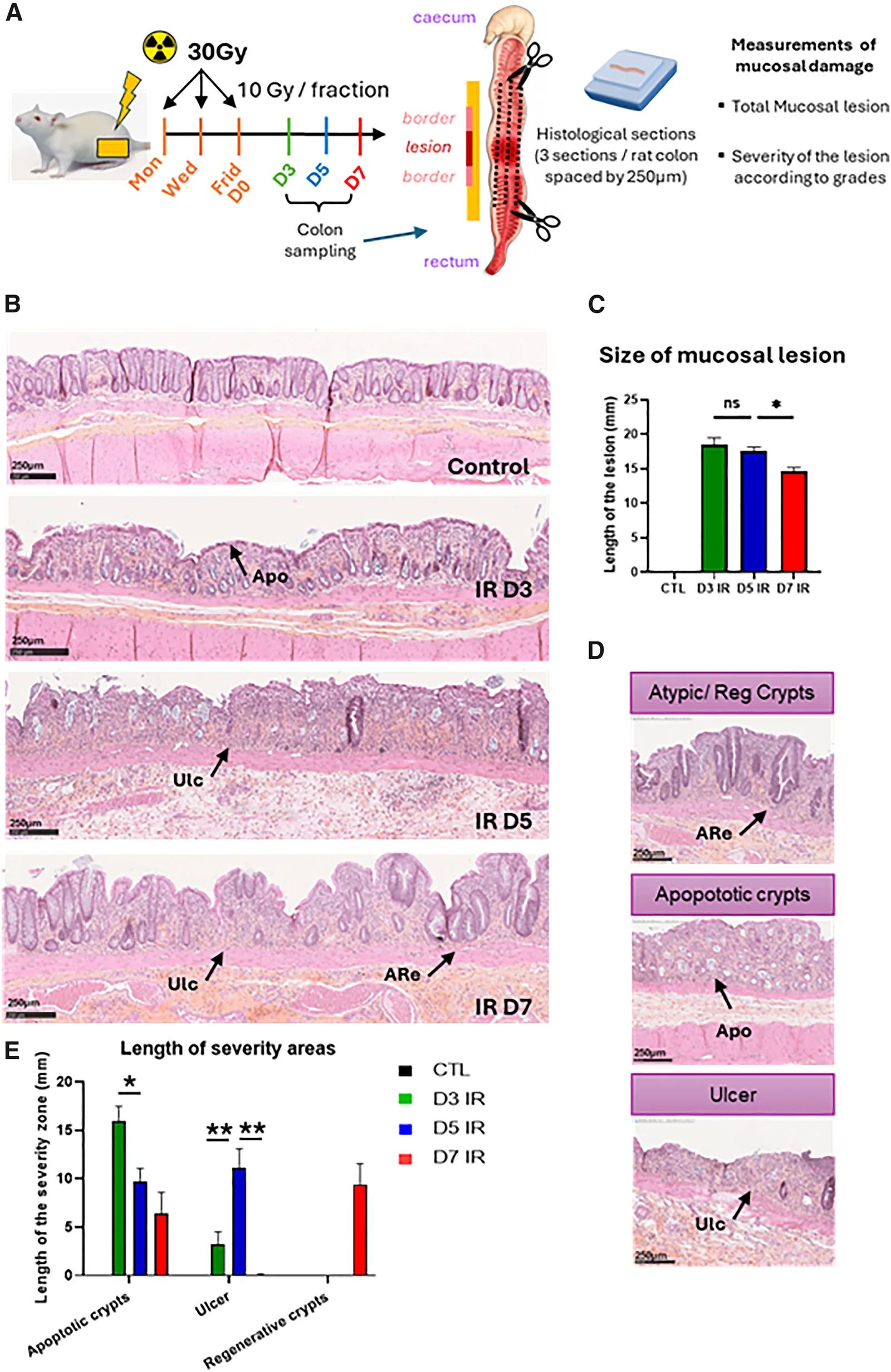
Colonic histological damage induced by irradiation delivered in 3 fractions and locally to the colon (A) Schematic representation of localized irradiation of the rat colon (irradiated area shown in yellow), including the timeline of the fractionated irradiation protocol and the time of tissue collection. A diagram of the colon opened longitudinally is shown, with the caecum and rectum indicated. Irradiation-induced lesions are depicted in dark red, with adjacent border zones in pink. Dashed lines indicate the three series of sections collected at 250 μm intervals. (B) Representative images of colorectal tissue sections stained with hematoxylin-eosin-saffron in control and irradiated rats at days 3, 5, and 7 after the final irradiation fraction. Arrows indicate the different types of lesions. Scale bars, 250 μm. (C) Quantification of total mucosal lesion length. (D) Representative images illustrating the different lesion severity grades used for quantification. Scale bars, 250 μm. (E) Histogram showing the length of each damaged zones according to severity (± SEM). Data represents *n* = 5–6 per group and with values averaged from three section series per animal. Error bars represent SEM. Shapiro-Wilk normality test is followed by Kruskal-Wallis statistical test. ∗*p* < 0.05, ∗∗*p* < 0.01. Apo, apoptotic crypts; Are, atypic or regenerative crypts; Ulc, ulcer.

To establish a relevant animal model of radiation proctitis, rats were exposed to a total local radiation dose of 30 Gy, administered to the colon in three fractions of 10 Gy each. Histological analyses were conducted at days 3, 5, and 7 post-irradiation to characterize the progression of acute colonic injury (Figure 1A). We assessed both the total length of the lesion and the severity of tissue damage in histological sections over time. Mucosal lesion length was quantified and compared with non-irradiated control animals (Figures 1B and 1C). In control rats, the colon exhibited uniformly distributed crypts above the muscularis mucosa, oriented toward the lumen. The irradiation protocol induced significant epithelial injury, with the longest mucosal lesions observed as early as day 3. Lesion length remained elevated on day 5 but decreased significantly by day 7 (Figure 1C).

To further evaluate lesion severity over time, we performed a detailed histomorphological analysis of epithelial alterations following acute irradiation. Three distinct histological structures were defined based on morphometric criteria (Figure 1D). *Apoptotic crypts* were identified by epithelial cell depletion within the crypts, accompanied by intraluminal necrotic debris, resulting in degenerating or necrotic crypts. *Atypical (regenerative) crypts* were characterized by highly proliferative epithelial cells, glandular distortion, and abnormal orientation relative to the colonic lumen. The most severe mucosal injury, defined as *ulcer*, corresponded to a near-complete loss of crypt structures and absence of lining epithelium.

Localized irradiation induced the appearance of apoptotic crypts by day 3, progressing to ulceration by day 5 (Figures 1B–1E). By day 7, crypts were sparse, and atypical crypt structures enriched with proliferative epithelial cells were detected within the irradiated area, indicating the initiation of epithelial regeneration (Figures 1B–1E). Collectively, these histological analyses demonstrate that both the maximal extent and severity of mucosal injury occur at day 5 following fractionated local irradiation of the colon.

To assess the relevance of our rat model in reproducing chronic damage after RT, we examined colonic tissue at a later time point (day 62). Histological analysis revealed that the epithelial mucosa was restored at this stage. However, persistent tissue alterations were evident, including inflammatory features associated with crypt detachment from the muscularis mucosa and distension of the muscular layer (Figure S1). Additionally, fibrosis was detected using picrosirius staining (Figure S2).

### Innate inflammatory cell infiltration following irradiation

We next evaluated inflammatory cell infiltration by quantifying CD68^+^ macrophages and MPO^+^ neutrophils in colon sections using immunohistochemistry (IHC). Quantification was performed in both the mucosa and submucosa (Figure S3).

**Figure 2:**
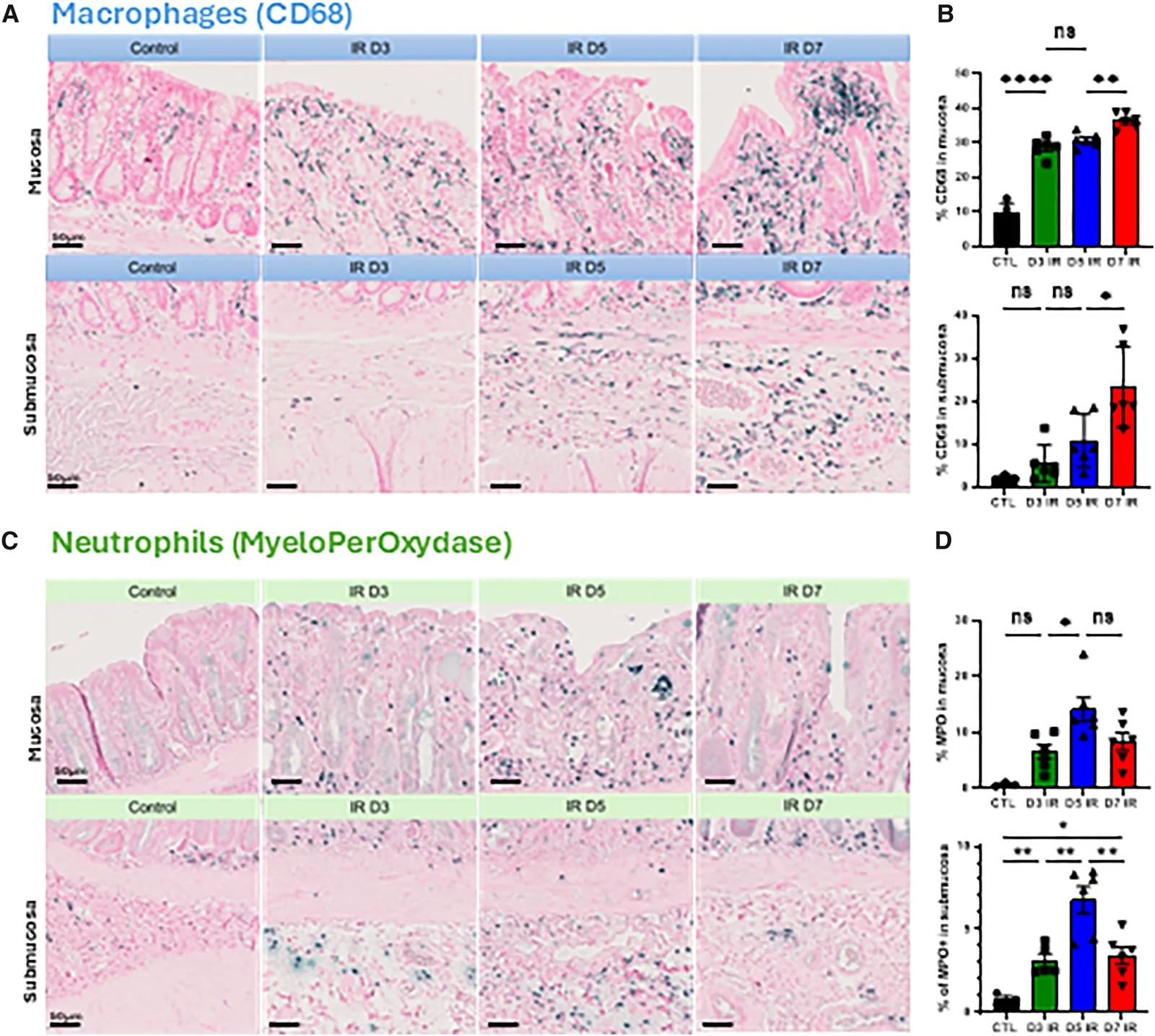
Acute innate inflammatory cell infiltration in the colonic mucosa and submucosa following irradiation (A) Representative pictures of CD68 immunohistochemistry in control and irradiated rats at day 3, 5, and 7 after the last fraction of irradiation. (B) Quantification of the percentage of CD68-positive cells out of the total number of cells. (C) Representative pictures of myeloperoxidase (MPO) immunohistochemistry in control and irradiated rats at day 3, 5, and 7 after the last fraction of irradiation. (D) Quantification of the percentage of MPO positive cells out of the total number of cells. All quantifications were performed using QuPath. (*n* = 5–6 per group; Error bars represent SEM. One-way ANOVA statistical test. ∗*p* < 0.05, ∗∗*p* < 0.01, ∗∗∗∗*p* < 0.0001. Measurement bars, 50 μm.

In control tissue, resident macrophages were predominantly localized in the mucosa rather than the submucosa (Figures 2A and 2B). At day 3 post-irradiation, the number of macrophages significantly increased in the mucosa (*p* < 0.001) and remained elevated at day 5. In the submucosa, macrophage infiltration became significantly different from control levels at day 5 (*p* < 0.05). By day 7, macrophage infiltration further increased in both compartments (day 5 vs. 7: *p* < 0.01 in the mucosa and *p* < 0.05 in the submucosa).

Neutrophils (MPO^+^) infiltrate in the mucosa and submucosa in similar temporal pattern (Figures 2C and 2D). In the mucosa, MPO^+^ cell counts increased at day 3 post-irradiation, peaked at day 5 (Figure 2C), and then decreased by day 7 (*p* < 0.05) (Figure 2D), although infiltration remained above control levels.

### Molecular inflammatory response to irradiation

**Figure 3:**
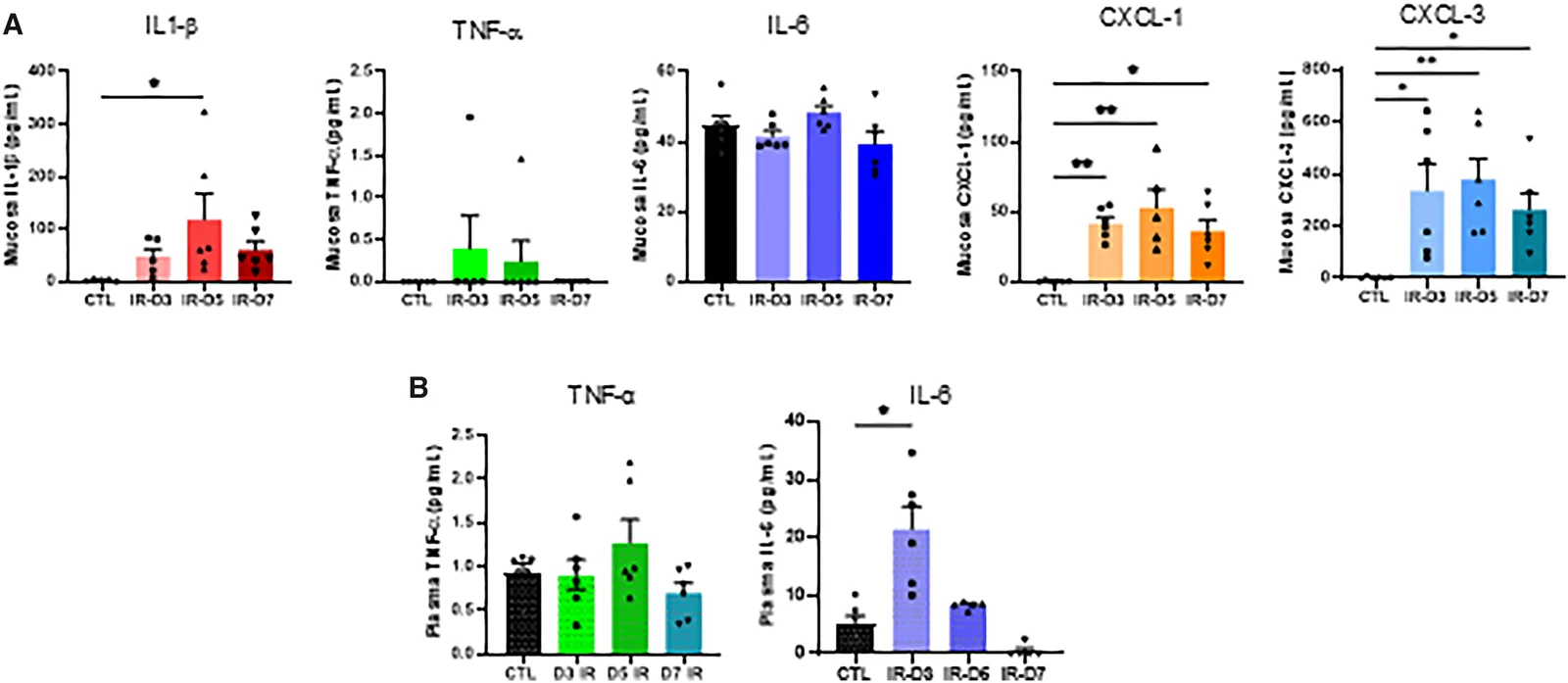
Localized and fractionated irradiation induced inflammatory molecules in colonic mucosa and plasma (A) Histograms showing cytokines or chemokines levels detected by ELISA on colonic mucosal tissues. (B) Histograms showing cytokines levels detected by ELISA in blood. Histograms are obtained with *n* = 5–6 per group. Error bars represent SEM. Shapiro-Wilk normality test is followed by Kruskal-Wallis statistical test. ∗*p* < 0.05, ∗∗*p* < 0.01.

To further characterize the inflammatory response, we measured cytokine and chemokine levels in colonic mucosal scrapings and plasma using ELISA (Figure 3). In mucosal samples, IL-1β levels increased by day 3 post-irradiation, peaked at day 5 (approximately 130 pg/mL, *p* < 0.05 vs. control), and subsequently declined, yet remained elevated compared with controls. Mucosal TNF-α exhibited a non-significant trend toward increased levels at days 3 and 5, whereas IL-6 levels were unaffected by irradiation. The neutrophil chemoattractants CXCL1 and CXCL3 were significantly upregulated from day 3 to day 7 post-irradiation (Figure 3A).

While IL-1β was significantly elevated locally in the irradiated colon, it was not detectable in rat plasma. Plasma TNF-α levels increased at day 5 post-irradiation, and plasma IL-6 was significantly elevated at day 3 (approximately 22 pg/mL, *p* < 0.01 vs. control) and day 5, before returning toward baseline by day 7 (Figure 3B).

Taken together, these findings demonstrate a predominantly localized production of pro-inflammatory mediators following fractionated irradiation, with only a transient systemic inflammatory response detectable at early time points (days 3 and 5).

### Fabrication and characterization of the bilayer films for drug delivery

**Figure 4:**
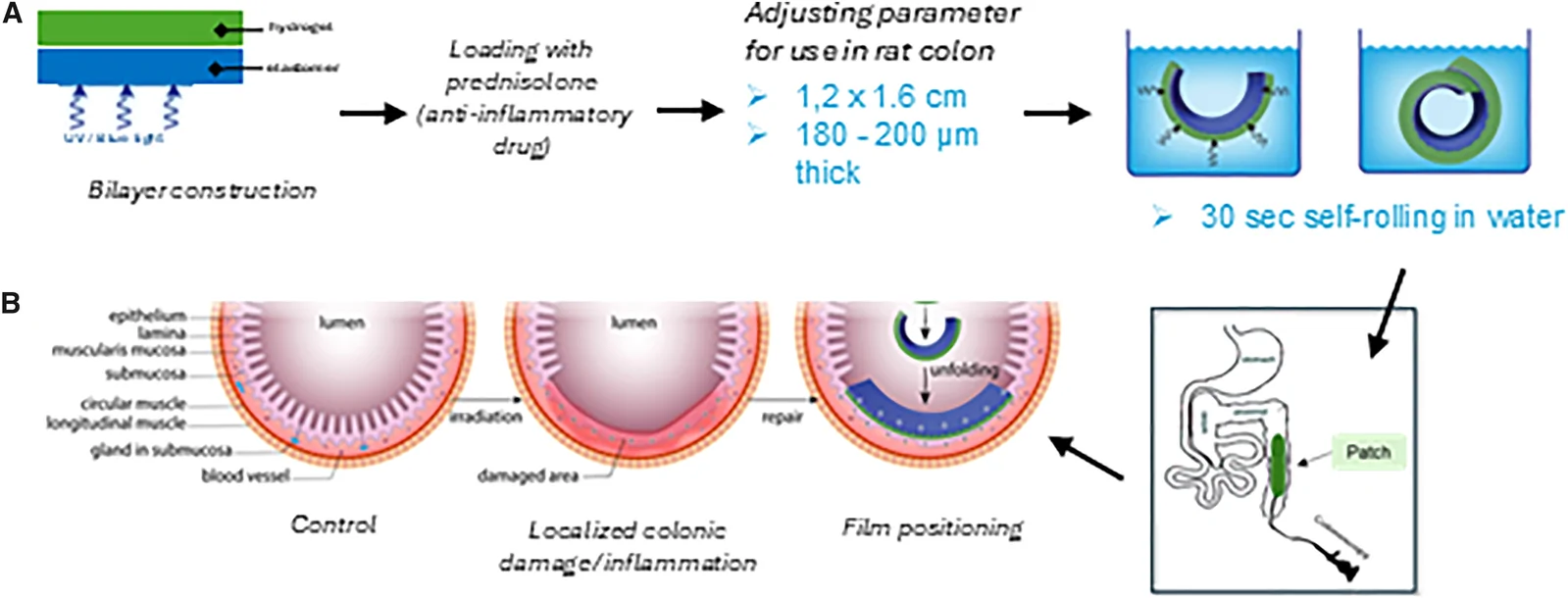
Set up of self-rolled device for intraluminal deposition under endoscopy (A) Method for manufacturing and adapting self-rolling devices for endoscopic use. (B) Scheme of the colon in control condition, after localized irradiation and after film positioning on irradiated zone.

According to previous studies,^22^^,^^23^ we manufactured a self-rolled bilayer patch designed for endoscopic intraluminal delivery to target localized colonic inflammation. The bilayer films were prepared using a 2-steps simple casting method (Figures 4A and 4B). (PEG_10k_-PLA_69k_)_8_ (hydrophobic layer noted H^−^L) polymer solution was poured in the aluminum cup mold and was let evaporate slowly (polyethylene glycol [PEG] and hydrophobic polylactic acid [PLA]). Then, the (PEG_20k_-PLA_13k_)_8_ (hydrophilic layer noted H^+^L) precursor solution was poured onto the dry H^−^L layer and was also let to evaporate slowly. The resulting polymer bilayers were UV-photo crosslinked then immersed in prednisolone solution in ethanol to load the anti-inflammatory molecule. Upon immersion in water, the film automatically rolled into a cylindrical shape and was then endoscopically implanted into the irradiated, inflamed colon of the rat (Figures 4A and 4B).

**Figure 5:**
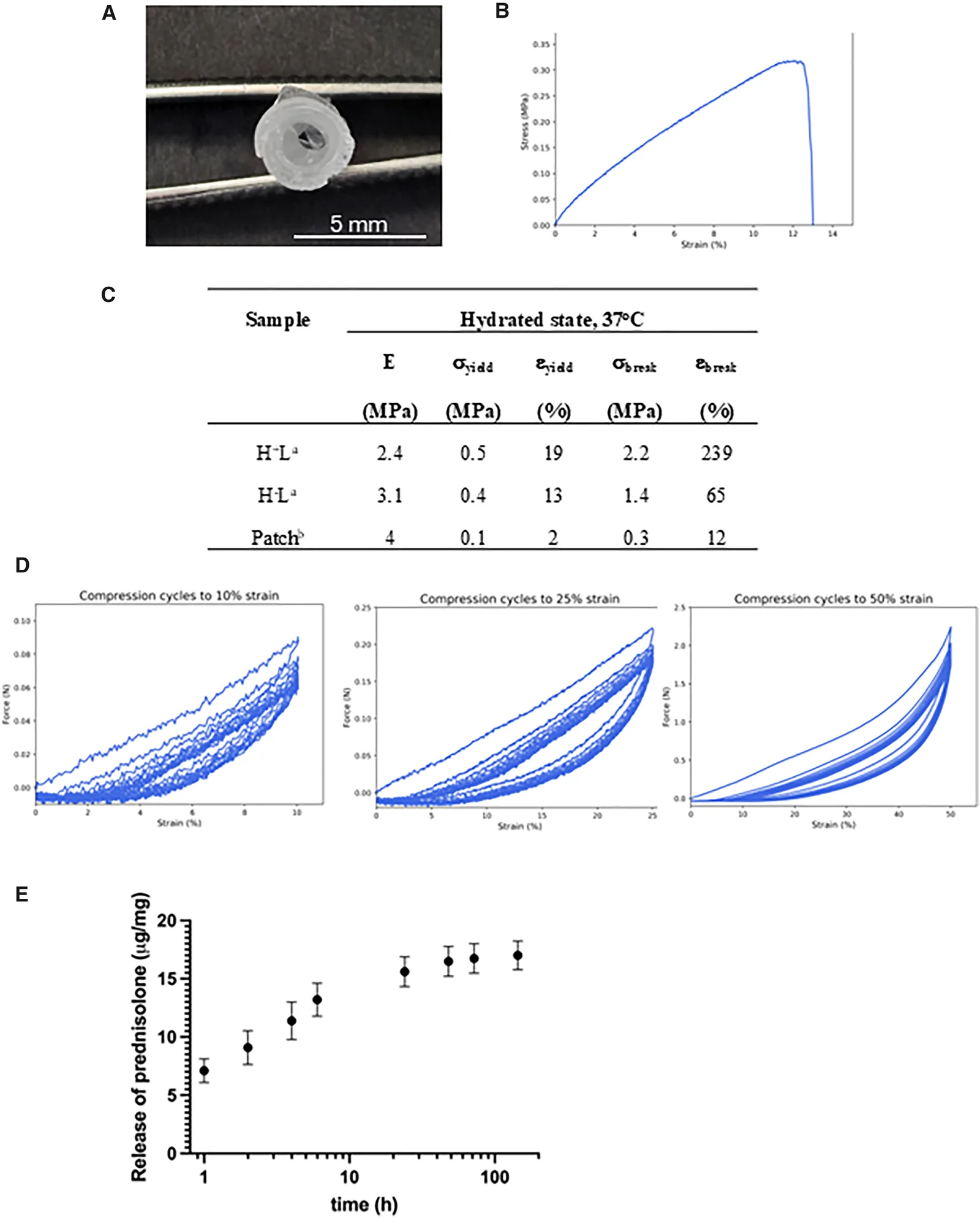
Mechanical properties and drug delivery profiles of the self-rolled device (A) Picture of the bilayered construct (cross section) after immersion and swelling in water. (B) Mechanical evaluation of the bilayered patch by tensile tests. Stress-strain curves obtained in the hydrated state at 37°C. (C) Table depicting mechanical properties in the hydrated state at 37°C of the individual constitutive layers and bilayers. (a) Values tested on individual layers on dog-bone sample shapes previously measured in.^24^ (b) Values tested on a strip of unrolled hydrated patch (see details in the experimental model and study participant details). (D) Mechanical evaluation of the bilayered patch under cyclic compression loads at 10%, 25%, and 50% strain. (E) Normalized prednisolone release kinetics. *In vitro* cumulative release profile in PBS (37°C) for films loaded with 60 mg of drug in ethanol. Data are normalized to polymer mass, illustrating a sustained delivery profile. Values are presented as means ± SD (*n* = 3).

The mechanical properties of the individual layers and the bilayered patch were assessed through tensile and compression tests performed in the hydrated state at 37°C (Figure 5A).

Tensile tests (Figures 5B and 5C) performed on hydrated strips excised from the patch revealed that the bilayered construct exhibits a higher elastic modulus (4 MPa) compared to each individual layer. In contrast, the bilayered material displayed reduced elongation at break (12%), together with lower strain and stress at failure, relative to the single-layer counterparts. At first glance, these features could suggest a potential limitation in terms of mechanical robustness during handling.

However, it should be emphasized that the functional configuration of the patch is a self-rolled, multilayered cylindrical construct (Figure 5A), which is not designed to undergo tensile deformation. Instead, implantation involves mechanical compression using forceps prior to insertion into the colon. Accordingly, cyclic compression tests were conducted at 10%, 25%, and 50% strain to assess the structural recovery following deformation. The compression-release profiles exhibit a marked hysteresis (Figure 5D), indicating a non-ideal elastic response. Nevertheless, the construct withstands repeated compressive loading and demonstrates efficient shape recovery, returning to its initial cylindrical geometry with limited residual strain (<10%), even at high levels of deformation. This behavior is a critical parameter ensuring adequate manipulability and facilitating the implantation procedure.

To optimize the therapeutic payload, prednisolone, which exhibits limited aqueous solubility (0.23 mg/mL), was dissolved in ethanol (33.33 mg/mL) to prepare highly concentrated loading solutions. Dry films were immersed in an ethanolic prednisolone solution (60 mg) for 24 h at 37°C, allowing for maximal drug entrapment within the matrices.

The release profiles were subsequently evaluated in 10 mL of PBS (1×, pH 7.4) at 37°C and quantified via reverse phase high-performance liquid chromatography (RP-HPLC). Analysis of the release kinetics revealed that 50% of the payload was discharged within approximately 2 h, with 90% release achieved at the 24-h mark (Figure 5E). In a previous article, we have determined that the drug release is governed by a Fickian diffusion mechanism.^22^ Using a loading mass of 60 mg, the total amount of prednisolone released in the medium is estimated to 17.5 μg/mg of polymer.

### Optimized self-rolled film for deposition by endoscopy and retention in the colon

To facilitate endoscopic deployment in the rat colon, we optimized the film dimensions to 1.6 × 1.2 cm in surface area and 180–200 μm in thickness. At these specifications, the film rapidly self-rolled upon immersion in water (within ∼10 s), enabling easy grasping with biopsy forceps and compatibility with standard endoscopic procedures.

**Figure 6:**
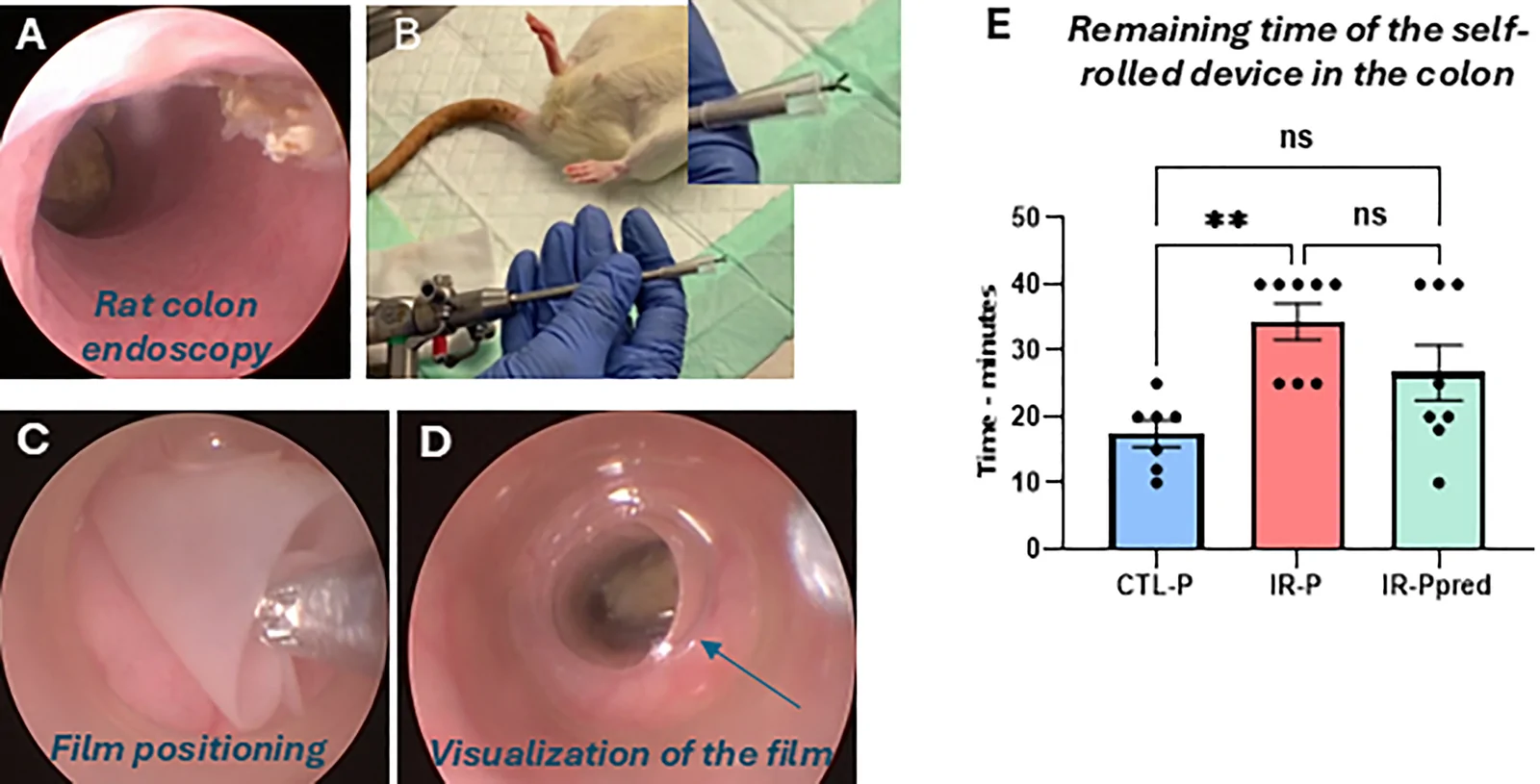
Placement of the self-rolled device in the rat colon (A) Endoscopic pictures showing the normal rat colon. (B) Picture of the forceps used to place the self-rolled device. (C) Pictures showing the forceps that hold the patch in place at the desired location in the colon. (D) Picture visualizing the self-rolled device once placed in the colon. (E) Quantification of the self-rolled device retention time in the colon (*n* = 7–8 rats per group) in normal condition. Error bars represent SEM. Shapiro-Wilk normality test is followed by Kruskal-Wallis statistical test. “ns” no significance. ∗∗*p* < 0.01. CTL-P, control patch, IR-P, irradiated ppatch, IR-Ppred, irradiated patch charged with prednisolone.

The film was then deployed onto the colonic mucosa via colonoscopy, specifically targeting inflamed areas (Figures 6A–6D; Video S1). We first assessed film retention time in both control and irradiated animals (Figure 6E). In non-irradiated controls (CTL-P), patches remained in place for an average of 17 min. In irradiated animals (IR-P), retention time significantly increased to 34 min (*p* < 0.01 vs. CTL-P), suggesting that irradiation-induced mucosal alterations may enhance patch adhesion or reduce peristaltic activity.

Video S1. Self-rolled film positioning *in vivo*, related to Figure 3

To further extend retention and prolong prednisolone release, we tested two interventions: prolonged anesthesia (20 min) and administration of Robinul V, a peripheral anticholinergic agent known to inhibit colonic peristalsis. In control animals, Robinul V treatment significantly increased patch retention from 28 min (without treatment) to 46 min, within this time frame, the patch was found to release 40% of its prednisolone payload in PBS, corresponding to approximately 360 μg. It should be noted, however, that the limited fluid availability in the colon (semi-dry conditions) presents a significant challenge for extrapolating exact release quantities *in situ*.

These results demonstrate the *in vivo* feasibility of the approach, including successful colon visualization, precise patch deposition using biopsy forceps, and confirmation of mucosal placement. Moreover, we established procedural conditions—such as the use of Robinul V—that ensure sufficient retention time for effective drug delivery to the colon.

### Effect of self-rolled device loaded with prednisolone on inflammatory markers

**Figure 7:**
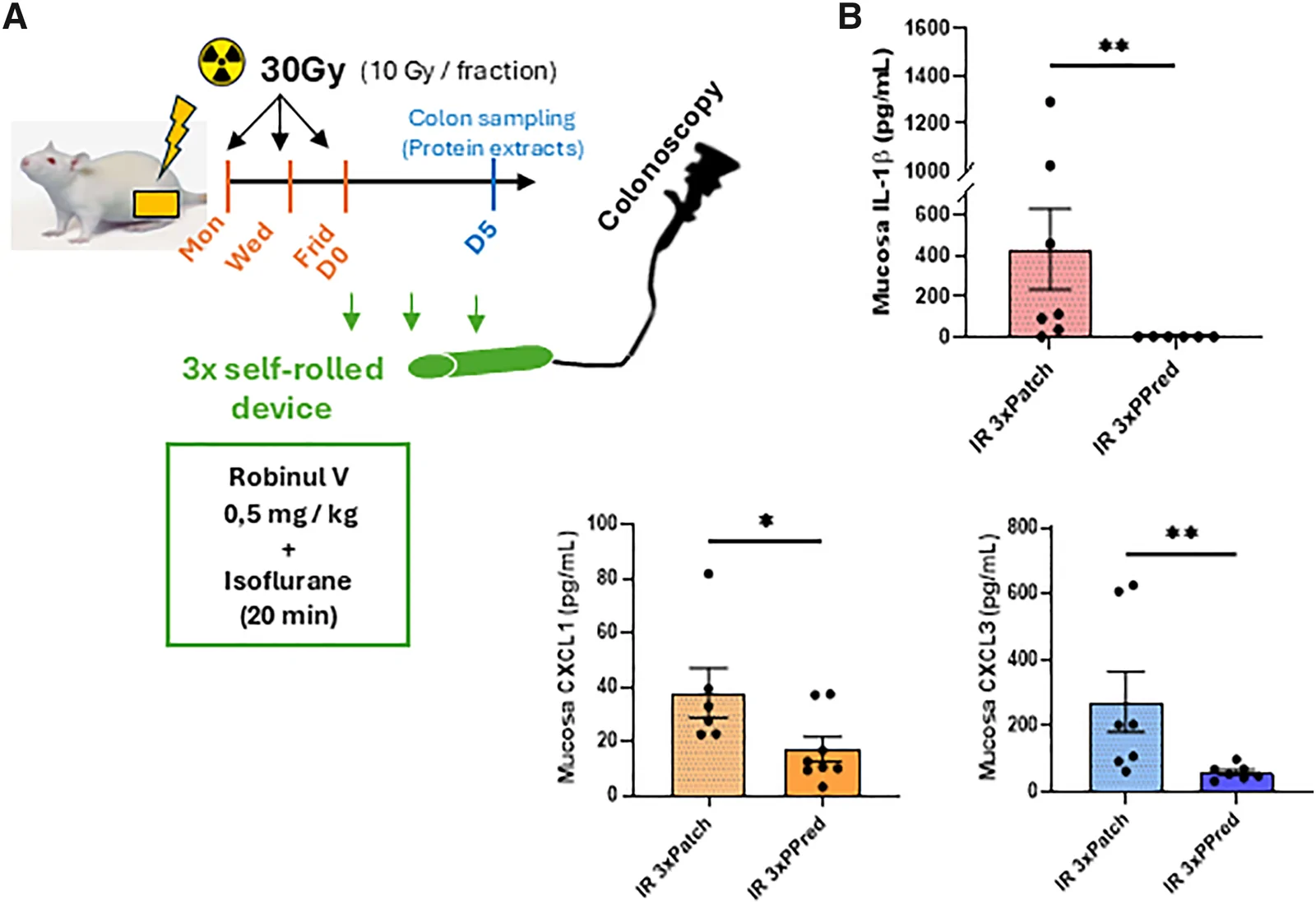
The *in vivo* effects of a self-rolled prednisolone-loaded device, endoscopically delivered, on inflammatory mediators in irradiated colonic mucosa (A) Schematic timeline of the experimental protocol, including fractionated irradiation and endoscopic deployment of the device using an optimized procedure. Prior to endoscopy, rats received an injection of Robinul V, followed by 20 min of anesthesia. (B) Quantification of cytokines and chemokines in the colonic mucosa of irradiated rats, measured by ELISA. Animals were treated with devices either loaded with prednisolone (PPred) or unloaded devices (Patch). Data represent *n* = 6–8 rats per group. Error bars represent SEM. Shapiro-Wilk normality test is followed by unpaired *t* test statistical test. ∗*p* < 0.05, ∗∗*p* < 0.01.

To evaluate the *in vivo* anti-inflammatory effect of prednisolone delivered via the self-rolled patch, we employed a rat model of irradiation-induced colonic inflammation (Figure 7A). Analyses were conducted at day 5 post-irradiation, corresponding to the previously established peak of inflammation.

Local treatment with the prednisolone-embedded patch significantly reduced mucosal levels of key acute inflammatory mediators, including IL-1β, CXCL1, and CXCL3 (Figure 7B). However, IL-6 and TNF-α levels remained unchanged, and no systemic modulation of inflammation was observed, as confirmed by plasma IL-6 measurements via ELISA.

## Discussion

The onset of radiation proctitis can significantly impair patients’ quality of life and, in severe cases, may even necessitate treatment discontinuation. Prostate cancer, the most frequent cancer in men, has seen a rising incidence primarily due to population growth, aging, and improved detection methods.^24^ While cancer treatment is tailored to each patient, approximately 30% of prostate cancer patients undergo RT. Currently, the incidence of RT-induced side effects (all grades) is estimated at 5% five years post-treatment, increasing by 1% per year (reaching 10% ten years after RT, and so on)^25^ Given that prostate cancer is now cured in 98% of cases, the growing number of cancer survivors heightens the risk of developing RT-related side effects. Therefore, developing preventive treatments for radiation proctitis, rather than addressing it after onset, could significantly enhance patients’ quality of life.^26^

In this study, we employed a localized and fractionated irradiation model in rats to induce colonic damage. This model resulted in acute epithelial injury, manifesting as ulcers spanning 12 mm in length by day 5. The irradiation doses and targeted volume allowed for epithelial regeneration from healthy margins, although regenerative crypts were also observed within the ulcerated areas on histological sections. Over time, epithelial coverage was improved, yet persistent inflammation was evident, as demonstrated by colonic muscle distension and submucosal edema. This chronic, low-grade inflammation aligns with clinical observations in irradiated patients. A notable study examining mucosal biopsies from irradiated survivors revealed chronic inflammation, bacterial infiltration, and neutrophil activity,^6^ findings that are entirely consistent with our rat model. However, unlike the 1%–2% of patients who develop severe lesions such as obstruction, our study did not observe such complications, potentially due to the limited number of animals included.

An optimal drug therapy must balance efficacy with minimal side effects. Localized delivery of active compounds directly to injured tissues ensures targeted efficacy while reducing systemic adverse effects. Although numerous oral formulations for colonic drug delivery have been developed, their implementation remains challenging due to significant drug absorption in the upper gastrointestinal tract, pH variations, and inconsistent intestinal transit times.^14^ While intrarectal administration can achieve higher local tissue concentrations compared to oral routes, its efficacy is highly variable due to differences in retention time, particularly in patients with impaired rectal compliance. Our study aims to innovate in the field of biomaterials by enabling precise and effective drug delivery to inflamed colonic sites, while ensuring practicality in clinical settings.

Previously, we developed self-rolling tubes using biodegradable and cytocompatible materials^23^ capable of delivering prednisolone, a potent anti-inflammatory drug.^22^ To align with clinical standards, we utilized PEG and PLA polymers, both of which are listed in the US Inactive Ingredients Database (published by the Food and Drug Administration in July 2017). These block copolymers are also components of many implantable medical devices marketed in the US and Europe. Additionally, we conducted cell viability assays on mouse L929 fibroblasts following ISO 10993–12 recommendations, confirming the non-toxicity of our PEG-PLA copolymer bilayer patches.^23^

In this study, we demonstrated proof of concept that self-rolling patches release prednisolone, effectively reducing irradiation-induced colonic inflammatory markers *in vivo*. These findings open promising avenues for testing other anti-inflammatory agents that can be loaded into these copolymers. Currently, biotherapies targeting anti-integrins (e.g., natalizuma and, vedolizumab) or anti-TNF agents (e.g., infliximab and adalimumab) have shown efficacy in inflammatory bowel disease.^27^ However, systemic administration of these drugs often leads to widespread adverse effects and requires high doses to ensure sufficient delivery to the injury site. Loading these agents into self-rolling films could enhance efficacy, reduce costs, and minimize undesirable effects. Future experiments could also explore the incorporation of epithelial regenerative molecules, such as epidermal growth factor (EGF),^28^ KGF,^29^ or GLP-2.^30^ This device could additionally benefit patients suffering from severe colonic inflammatory diseases, the incidence of which continues to rise, particularly in Western countries and among children.^31^ Restoring the epithelial barrier against pathogens remains a significant challenge in bowel diseases.^32^

In our study, we unfortunately observed inflammatory effects due to film positioning during endoscopy in rats. Specifically, IL-1β levels in the irradiated mucosa at day 5 were 150 pg/mL, compared to 400 pg/mL in the mucosa with the film. This effect may be exacerbated in small animals due to the relative size of the colonoscopes used. Although we utilized a colonoscope specifically designed for small animals, the colon-to-colonoscope ratio is approximately 3.5 in humans but only 2.1 in rats. Therefore, it would be valuable to replicate these experiments in larger animals, such as pigs, where the anatomical ratio more closely resembles that of humans. Importantly, in clinical practice, this local treatment with self-rolled films would likely be administered during pre-existing endoscopic procedures, such as vessel cauterization for managing hemorrhage in radiation proctitis or planned biopsies as part of the clinical follow-up for ulcerative colitis.^33^

In conclusion, we optimized a self-rolling film loaded with an anti-inflammatory agent. This endoscopy-based drug delivery strategy has been validated *in vivo* in a rat model of colonic inflammation, demonstrating a reduction in inflammatory markers. Further optimization is required to meet clinical needs. This work paves the way for testing the benefits of self-rolling tubes loaded with other relevant molecules. Additionally, a more detailed investigation into the pathophysiological mechanisms of radiation-induced colitis—particularly the key processes that could be targeted locally to enhance epithelial healing—would be highly valuable.

### Limitations of the study

This study aimed to provide proof of concept for the feasibility of using a self-rolled patch for implantation and *in vivo* anti-inflammatory drug delivery. Additionally, we developed a clinically relevant animal model of severe radiation-induced damage, for which no curative treatment currently exists. However, further experiments are needed to investigate the pathophysiological mechanisms and identify innovative molecular targets that can be loaded onto the patch and delivered locally to enhance epithelial healing.

## Resource availability

### Lead contact

Requests for further information and resources should be directed to and will be fulfilled by the lead contact, Noëlle Mathieu (noelle.mathieu@asnr.fr).

### Materials availability

The self-rolled film is available upon reasonable request and after discussion from the lead contact.

### Data and code availability

• Data reported in this paper will be shared by the lead contact upon request.
• This paper does not report original code.
• Any additional information required to reanalyse the data reported in this paper are available from the lead contact upon request.

## Acknowledgments

We thank Yoann Ristic and Miray Razanajatovo from the irradiation platform facility (ASNR/LDRI). Delphine Denais-Lalieve (DVM, Dip. LASM) and the GSEA team for their very precious help for animal care. We thank Laurent Pieuchot for art design. This work is supported by the 10.13039/501100001665French National Agency for Research (OPENN ANR-19-CE19-0022-01). D.A.-S., M.G., and S.O. are supported by ANR.

## Author contributions

Conceptualization, B.N., K.A., A.P., F.M., and N.M.; methodology, B.N., K.A., A.P., and N.M.; investigation, D.A.-S., C.D., M.G., S.O., A.P., and N.M.; writing—original draft, B.N., K.A., A.P., D.A.-S., and N.M.; writing—review & editing, B.N., K.A., A.P., D.A.-S., and N.M.; funding acquisition, B.N., K.A., F.M., and N.M.; supervision, B.N., K.A., A.P., and N.M.

## Declaration of interests

The authors declare no competing interests.

## Declaration of generative AI and AI-assisted technologies in the writing process

During the preparation of this work the author(s) used ChatGPT and DeepL AI in order to improve English writing. After using this tool/service, the author(s) reviewed and edited the content as needed and take(s) full responsibility for the content of the published article.

## STAR★Methods

### Key resources table

**Table:** 

REAGENT or RESOURCE	SOURCE	IDENTIFIER
**Antibodies**		

Mouse Anti-rat CD68	Biorad	MCA341R; RRID: AB_322219
rabbit anti-human/rat MPO	Abcam	Ab9535; RRID: AB_3086778
Polymer HRP anti-mouse	Diagomics	D55-110
Polymer anti-rabbit-HRP	Diagomics	D13-110

**Chemicals, peptides, and recombinant proteins**		

Citrate Buffer	Diagomics	ZUCO028-500
PBS 1×	Gibco	1159047
cOmplete^TM^, Mini Protease Inhibitor	Roche	11836153001
KIT protein dosage	Sigma	QPBCA-1KT

**Critical commercial assays**		

ELISA IL-1β	Bio-techne	RLB00
ELISA CXCL1	Bio-techne	RCN100
ELISA CXCL3	Bio-techne	RCN200
ELISA IL-6	Bio-techne	R6000B
ELISA TNFα	Bio-techne	RTA00

**Experimental models: Organisms/strains**		

Sprague-Dawley rats	Janvier SA	Sprague Dawley

**Software and algorithms**		

Software image viewer and analysis	Hamamatsu	NDP Viewer software V2
Software image analysis	QuPath®	v0.4.3.
Statistical software	GraphPad Prism	GraphPad (version 9.0.0)

**Other**		

Colonoscopy	Karl Storz	Coloview murine
Medical Accelerator	Elekta	Medical Accelerator
Decloaking chamber	Eurobio Biocare Medical	NxGen
Lysis tubes	MP Biomedicals	Lysis matrix D
Tissue homogenizer	Bertin	Precellys® Evolution
Digital slide scanner	Hamamatsu	S60

### Experimental model and study participant details

All animal experiments were performed at the animal facilities of the Nuclear Safety and Radiation Protection Authority (ASNR, Fontenay-aux-Roses, France, registry n°C92-032-01) in strict compliance with European directives (86/609/CEE) and were approved by local ethical committee of the ASNR. The experimental protocol was submitted to the French national authorization platform and after approval was registered under the APAFIS #45500–2023102716278752 v2 P23-15. We have complied with all relevant ethical regulations for animal use. Albinos outbred Sprague Dawley male rats of 8 weeks-old were used (Janvier SA, Le Genest Saint Isle, France). They were fed with standard pellets, food and water available *ad libitum*. Rats submitted to irradiation were anesthetized by isoflurane inhalation, and 3 fractions of 10 Gy were delivered in 1 week every 2 days (Monday, Wednesday and Friday) through a 2 × 3 cm window centered on the colorectal region using a medical accelerator (Elekta synergy_6 MVp X-rays with a mean photon energy of about 1.5 MeV; 30 kA; the dose rate was about 2.5 Gy per min) (Elekta SAS France).

### Method details

#### Colonoscopy

Colonoscopy was performed to place the patch. Under general anesthesia with 2% isoflurane, rats were placed in dorsal decubitus on a heated operation table. A COLOVIEW murine endoscopy system (Karl Storz, Germany) was used with biopsy forceps to hold the patch in place. Self-coiled device with prednisolone was deposited three times on days 0, 3 and 5 after irradiation versus unloaded patch. Rats were euthanized at day 5, 3 h after deposition of the last patch. To extend time retention of the patch, rats were injected subcutaneously with Robinul V 0.5 mg/kg (glycopyrrolate) 15 min before colonoscopy procedure.

#### Histological and immunohistochemistry

Rats were euthanized at day 3, 5, 7 after the late dose of radiation. Colons were excised, opened longitudinally, and immediately fixed in 4% formaldehyde (n = 5–6 per group). Colons were laid on their side prior to paraffin embedding. Paraffin-embedded tissues were sectioned into 5 μm-thick serial sections using a rotary microtome (Leica Microsystems AG, Wetzlar, Germany). For each colon, three series of sections separated by 250 μm were collected to allow evaluation of mean lesion length across the entire organ. Sections were stained with hematoxylin–eosin–saffron (HES) or picrosirius for histopathological assessment of colonic damage. Whole-slide images were acquired using a NanoZoomer S60 digital slide scanner (Hamamatsu, Japan), and colonic damage was measured using NDP Viewer software (Hamamatsu, France).

For immunohistochemistry, the protocols were as follow. For CD68 labeling, sections were dewaxed and treated with proteinase K for 5 min at room temperature. A peroxidase inhibition was realized with methanol 3% peroxide hydrogen for 10 min at room temperature. Mouse anti-rat CD68 (Bio-Rad France # MCA341R) was applied for 1 h at 37°C then Polymer HRP anti-mouse (Diagomics France #D55-110) was incubated for 30 min at room temperature. We used permanent HRP green as substrate and counterstaining with nuclear fast red.

For MPO (myeloperoxidase) labeling, the same protocol was used except the antigenic unmasking done with 10 mM Citrate Buffer pH = 6.0 (Diagomics France #ZUC028-500) 15 min at 110°C in decloaking chamber NxGen (Biocare Medical, Eurobio, France). Primary antibody was rabbit anti-human/rat MPO (Abcam UK #Ab9535). Secondary antibody was Polymer anti-rabbit-HRP (Diagomics France #D13-110). Immunolabeling analysis was performed with QuPath (v0.4.3. Software for Bioimage Analysis).

#### Cytokines measurements by ELISA

Blood was removed by intracardiac puncture, the plasma was recovered and stored at −80°C. The collection of colonic mucosae was performed through scraping, frozen in liquid nitrogen and stored at −80°C. (n = 5–6 per group).

The proteins were extracted using Lysis buffer (10 mL PBS (Gibco #11590476) with 1 tablet cOmplete, Mini Protease Inhibitor (Roche #11836153001)). Colonic mucosa was lysed in lysis tubes (Lysis matrix D, MP Biomedicals) containing 600 μL of lysis buffer. Tubes were placed on the tissue homogenizer (Precellys Evolution, Bertin, France) and processed using a generic homogenization program with the following settings 2 × 30s at 6000rpm · Break 1 min · Cooling 4°C followed by a centrifugation of 3 min at 2655 g at 4°C. The obtained homogenate was then transferred and then centrifuged 10 min at 15000 g at 4°C. The obtained supernatants were stored at −20°C.

The protein dosage was carried out following the manufacturer’s procedure for the KIT QPBCA-1KT (Sigma, protein range: 0.5 to 30 μg/mL). Mucosal samples were diluted 250-fold and 500-fold. For ELISA of the mucosa, we followed the manufacturer’s instructions for using the TNFα, IL-6, IL-1β (#RLB00), CXCL1 (#RCN100) and CXCL3 (#RCN200) kits (Bio-techne, France). 50 μg of protein per sample were deposited. For ELISA on the plasma, we used the TNFα and Il-6 kits following the manufacturer’s recommendations (Rat IL-6 immunoassay #R6000B and Rat TNFα immunoassay #RTA00 by Bio-techne, France). Plasma was deposited pure.

#### Biomaterials

Self-rolling bilayer films, composed of PEG-PLA and impregnated with the anti-inflammatory agent prednisolone, were prepared according to previously established protocols.^22^^,^^23^ The bilayer architecture was produced by sequential casting of the precursor solutions into an aluminum mold (43 mm in diameter), ensuring complete evaporation of the solvent (dichloromethane) between each layer. To obtain the targeted thickness for the patch, each layer (H^+^L and H^−^L) was obtained by dissolving 200 mg of the corresponding hydrogel or elastomer precursor in 2.5 mL of dichloromethane. Photo-crosslinking was subsequently performed under UV irradiation (λ = 365 nm, I = 74 mW/cm^2^) for 10 min (5 min on each side). Patch were excised at the selected dimension of 1.6 × 1.2 cm from this bilayered film. To achieve therapeutic loading, the crosslinked bilayers were immersed in an ethanol solution containing 60 mg of prednisolone, facilitating drug uptake through equilibrium swelling of the polymer matrix.

The drug release profile in PBS (1×) at 37°C demonstrates a sustained delivery mechanism: approximately 50% of the encapsulated prednisolone is released within the first 2 h, reaching 75% after 6 h. Based on previous characterizations,^22^ the cumulative release after 24 h is estimated at 17.5 μg of prednisolone per milligram of polymer scaffold.

#### Tensile tests

For individual layers dog-bone strips (working area 10 × 2 mm) were stamped from polymer films. For hydrated patches, a strip (working area 40 × 10 mm) was cut from an unrolled patch. The samples thickness was measured with a micrometer. The mechanical properties were assessed at room temperature on hydrated samples with an Instron 3344 testing machine equipped with a 500 N load cell at a deformation rate of 10 mm min^−1^.

#### Cyclic loads on patch

Compression was applied to the hydrated patch with an Instron 3344 testing machine equipped with a 500 N load cell at a deformation rate of 10 mm min^−1^. Compression was applied to reach 10% strain for the patch, subsequently let to recover (0%), and immediately being stretched again to 10% (10 cycles). The same tests were repeated with deformation of 25% and 50%.

### Quantification and statistical analysis

Quantitative data were presented as means ± sem or SD when indicated and analyzed with GraphPad Prism software, version 9.0.0 (GraphPad, San Diego, CA). Statistical significance was defined as ∗*p* < 0.05, ∗∗*p* < 0.01, ∗∗∗*p* < 0.001, ∗∗∗∗*p* < 0.0001. The variables were tested with normality test (Shapiro-wilk test). For the comparison of 2 groups, depending on whether it followed a normal distribution, we continued with unpaired *t* test. Otherwise, we used Mann-Whitney test. The comparison of several groups, depending on whether it followed a normal distribution or not, was carried out by ordinary one-way Anova (Tukey) or Kruskal-Wallis test respectively.
