# Supplementary material for: Self-rolling film for in vivo drug delivery to limit colonic inflammation induced after radiation exposure
Source: iScience. 2026 Sep 3;29(9):117453. doi: 10.1016/j.isci.2026.117453 (PMC13571658; doi:10.1016/j.isci.2026.117453)
Supplement: Supplementary file 1 [file mmc1.pdf]

iScience, Volume *29*

Supplemental information

Self-rolling film for *in vivo* drug delivery to limit colonic inflammation induced after radiation exposure

Diego Amarante-Silva, Christelle Demarquay, Benjamin Nottelet, Mathilde Grosjean, Sidzigui Ouedraogo, Fabien Milliat, Karine Anselme, Arnaud Ponche, and Noëlle Mathieu

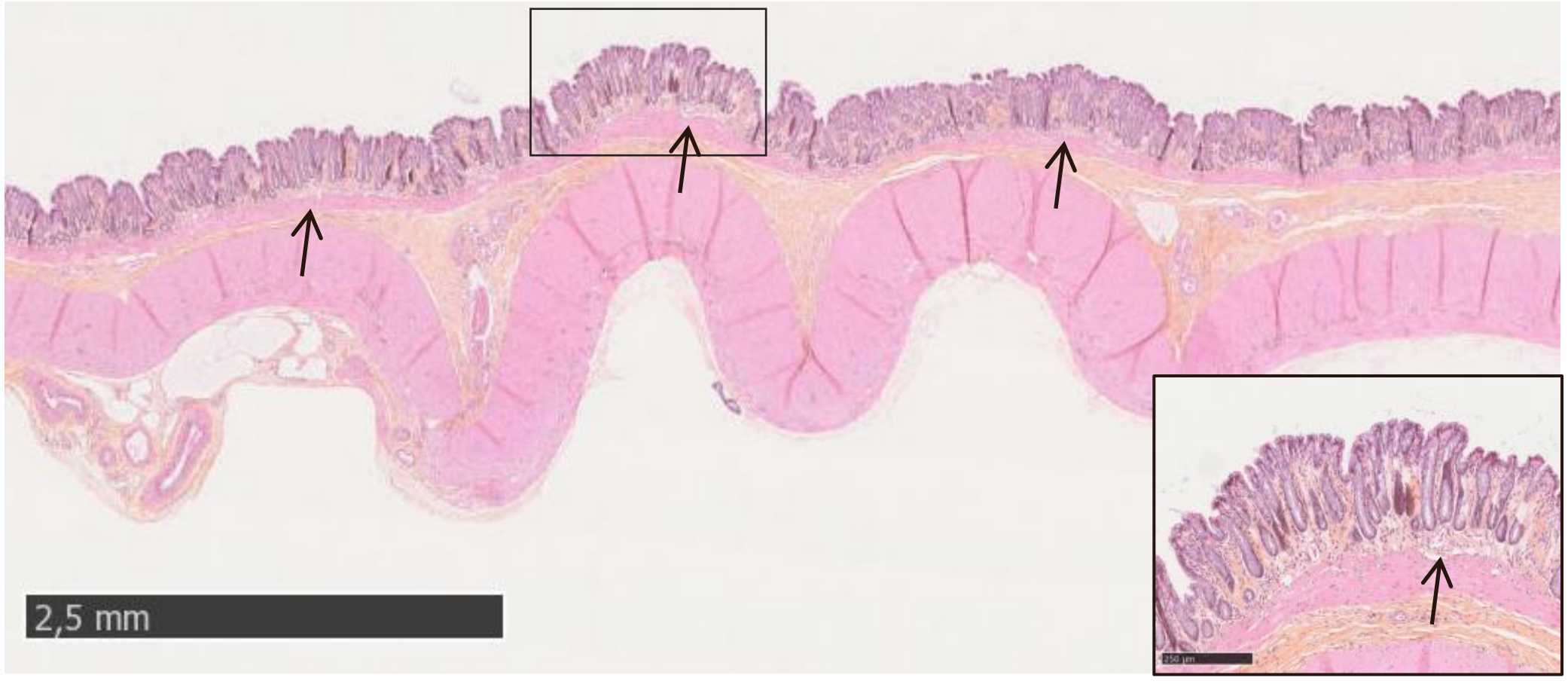

Suppl Figure 1 related to Figure 1: Representative pictures of colorectal tissues stained with hematoxylin-eosin-saffron, in irradiated rats at Day 62 after the last fraction of irradiation. Muscle distortion and crypts detachment from the muscularis mucosa are characteristic of inflammation (arrows). Scale bars represent 2.5mm or 250µm.

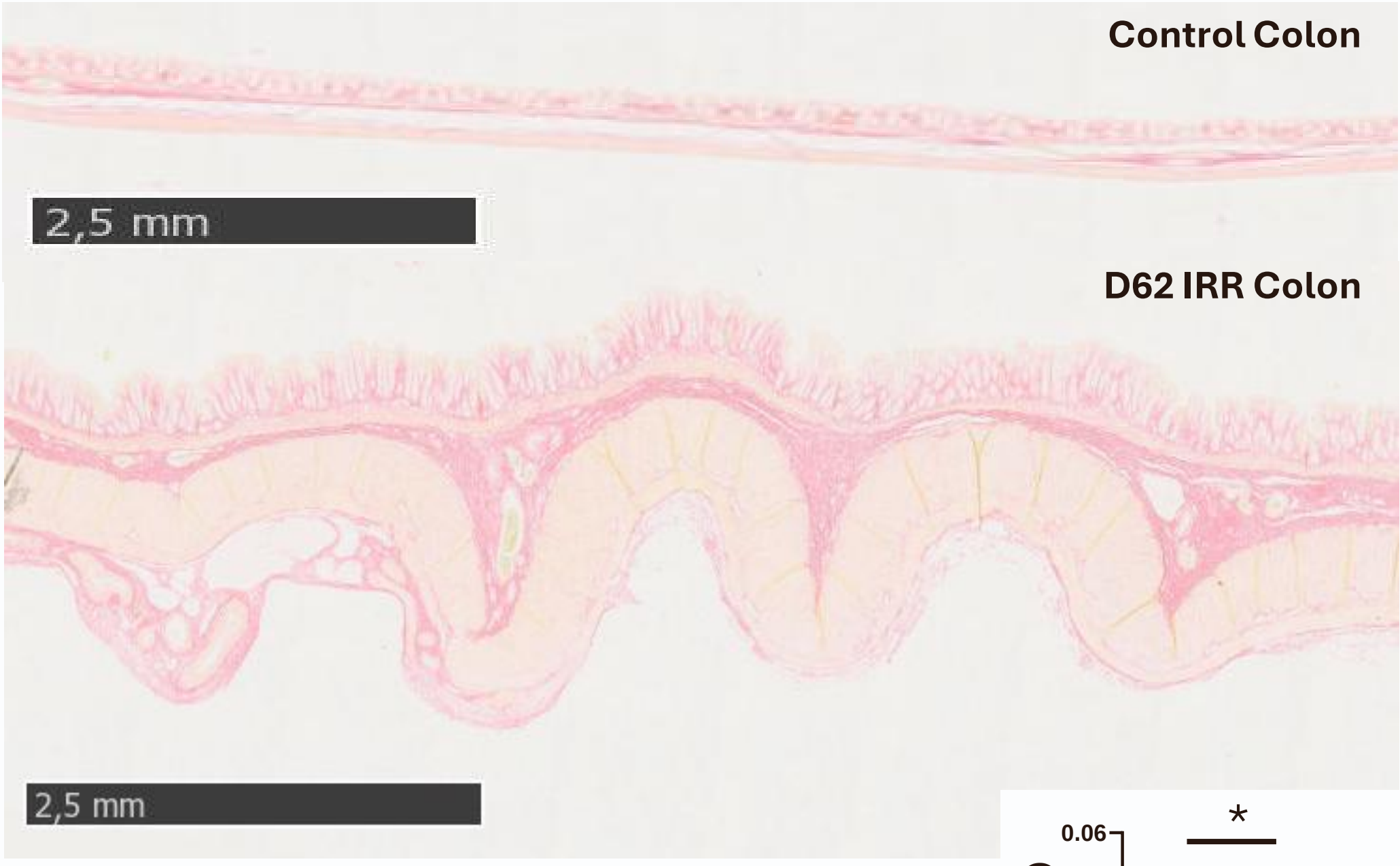

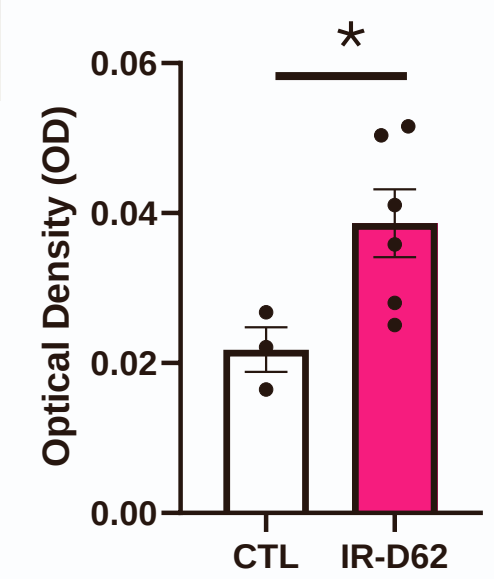

Suppl Figure 2 related to Figure1 : Representative pictures of colorectal tissues stained with picrosirius in control and irradiated rats colon at Day 62 after the last fraction of irradiation. This staining highlights fibrosis in irradiated colon compared to control colon. Collagen (in pink) deposition mainly accumulates in the submucosa. Scale bars represent 2.5 mm. Graph showing quantification using QuPath® software. Data represents n=5-6 per group and with values averaged from three section series per animal. Error bars represent SEM. Shapiro-Wilk normality test is followed by Kruskal-Wallis statistical test. * $p < 0.05$.

Mucosa

Sub-Mucosa

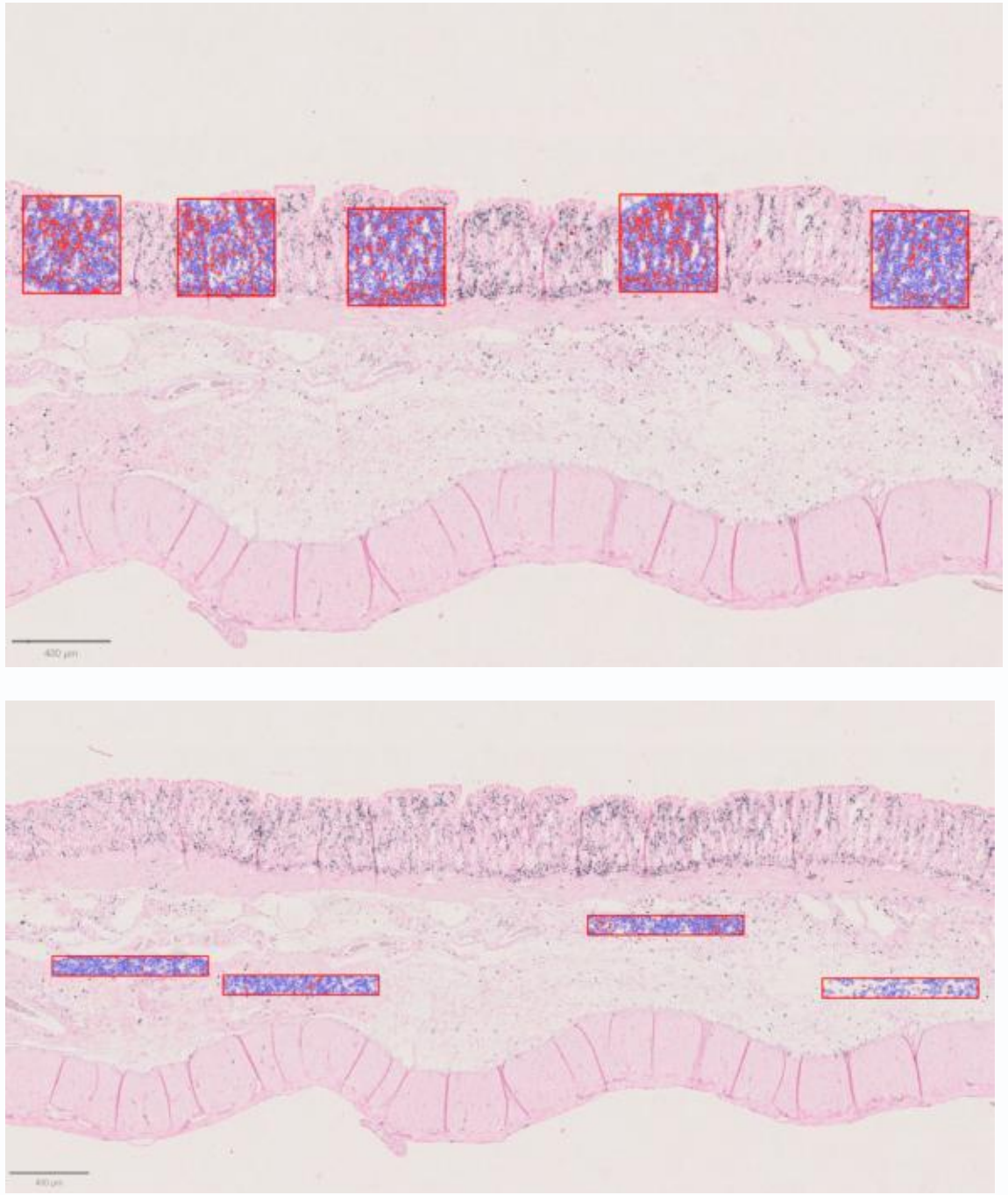

Suppl Figure 3 related to Figure 2 : Visualization of the quantified surface of the colon in the mucosa and the submucosa using QuPath®.
